# Supplementary figures and images for: CYRI-B-mediated macropinocytosis drives metastasis via lysophosphatidic acid receptor uptake
Source: eLife. 2024 May 7;13:e83712. doi: 10.7554/eLife.83712 (PMC11219039; doi:10.7554/eLife.83712)

Figure 2D

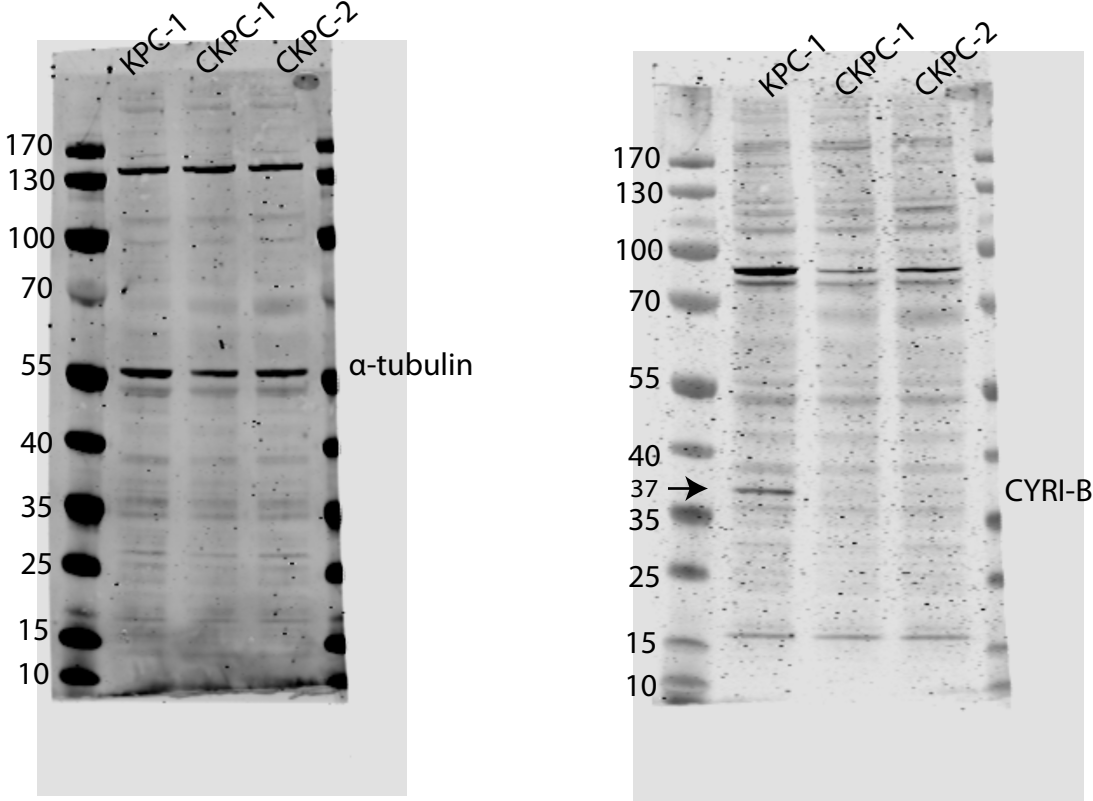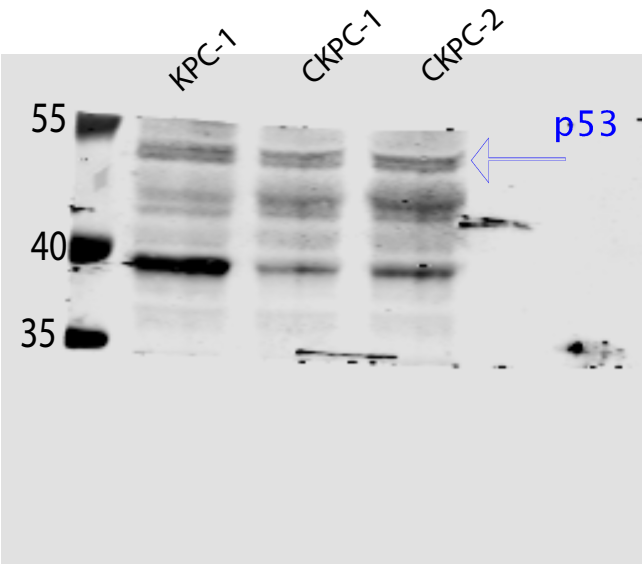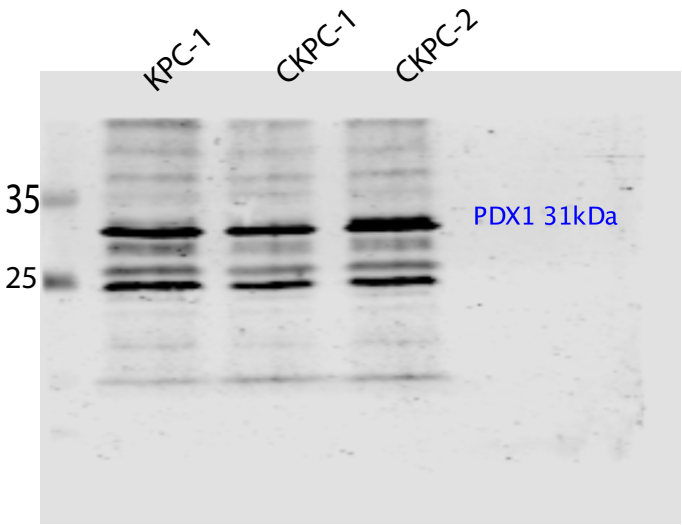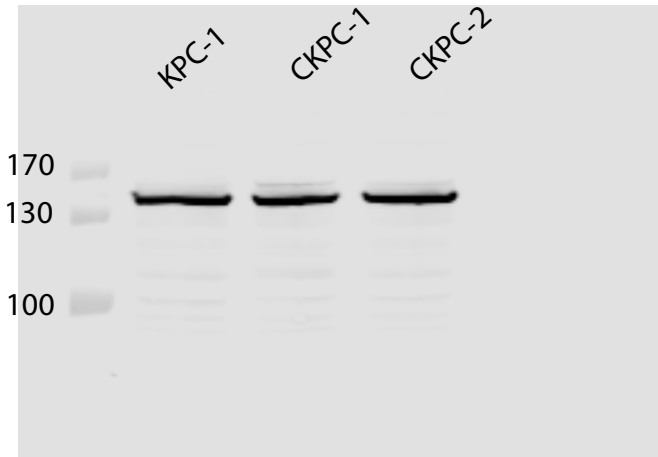

Supplement: Figure 2—source data 2. [file elife-83712-fig2-data2.zip › Figure 2- source data 2/labelled/Figure 2D-labelled.pdf]

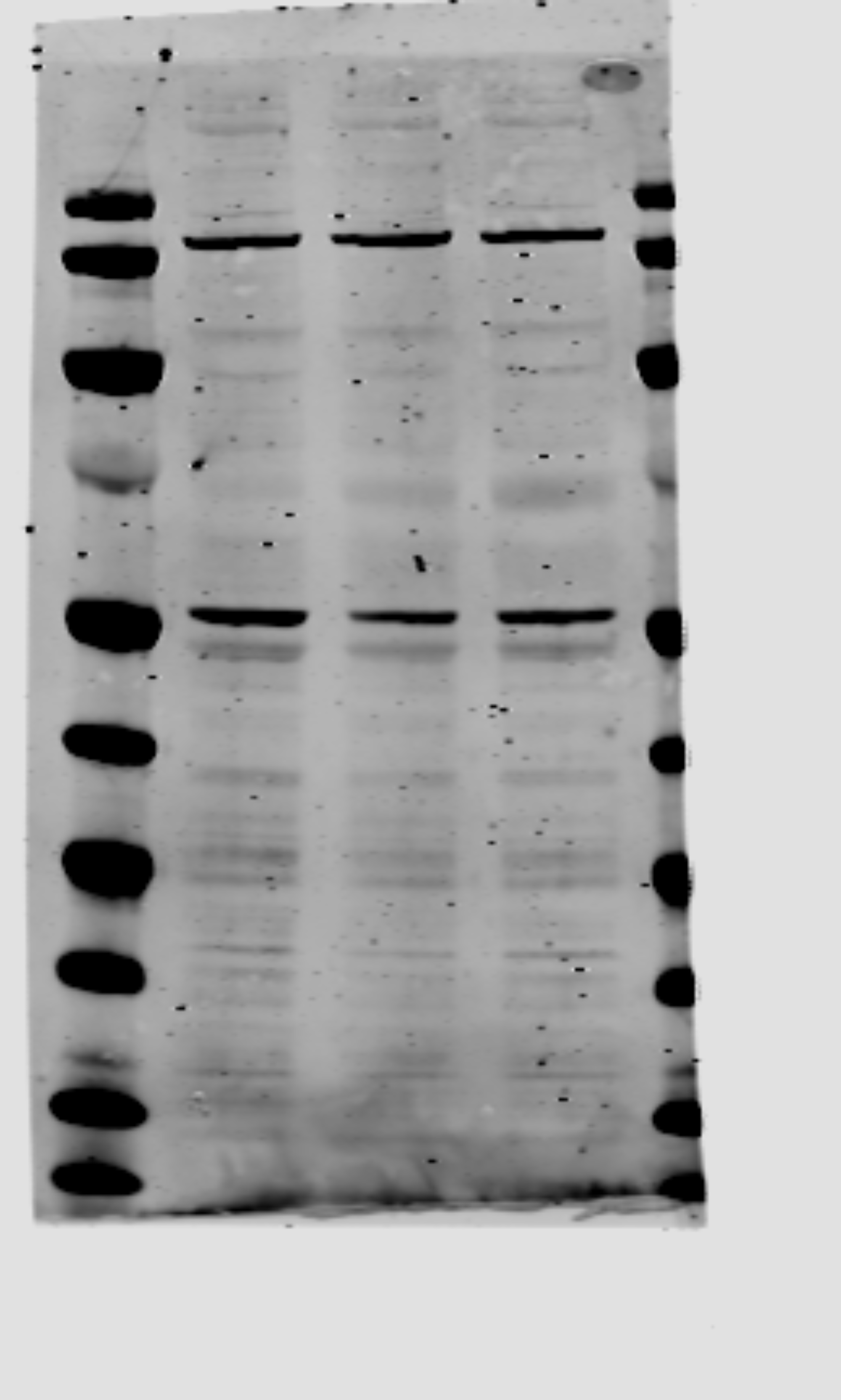

Supplement: Figure 2—source data 2. [file elife-83712-fig2-data2.zip › Figure 2- source data 2/unlabelled and uncropped/a-tub rep2 in PDACB and BSNA9.4a and 9.5a.tif]

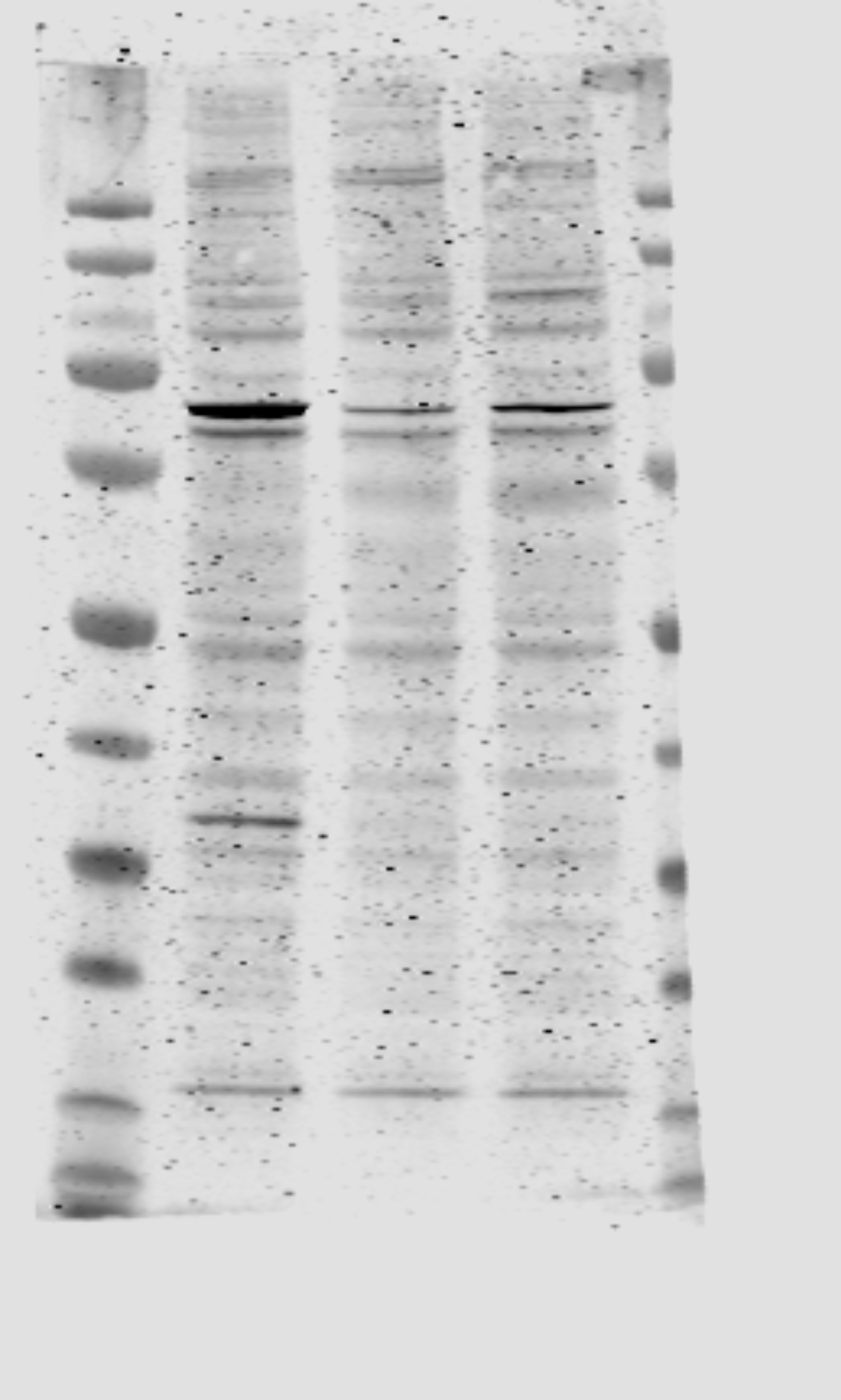

Supplement: Figure 2—source data 2. [file elife-83712-fig2-data2.zip › Figure 2- source data 2/unlabelled and uncropped/CYRI-B rep2 in PDACB and BSNA9.4a and 9.5a.tif]

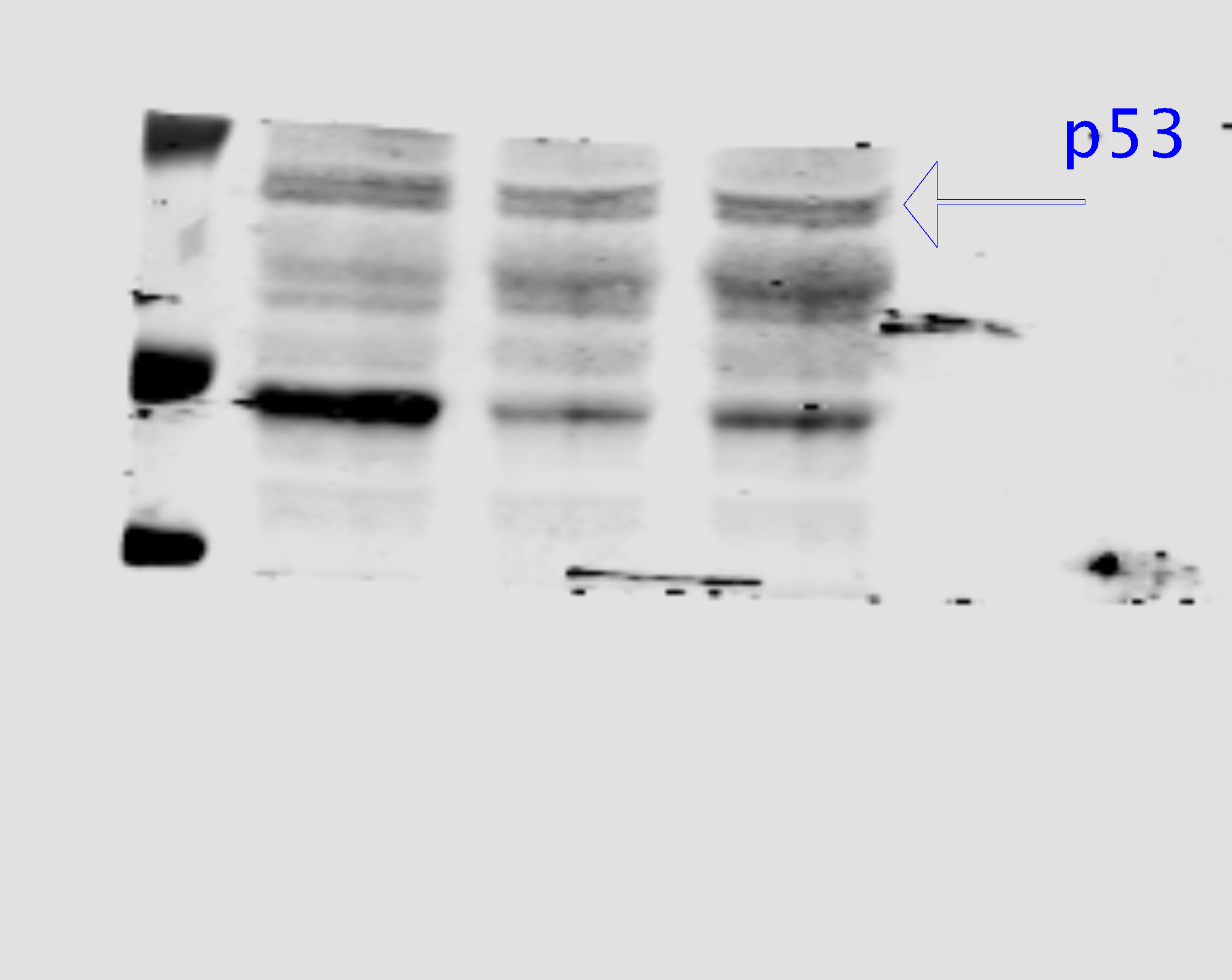

Supplement: Figure 2—source data 2. [file elife-83712-fig2-data2.zip › Figure 2- source data 2/unlabelled and uncropped/p53 rep2 in PDACB and BSNA9.4a and 9.5a.tif]

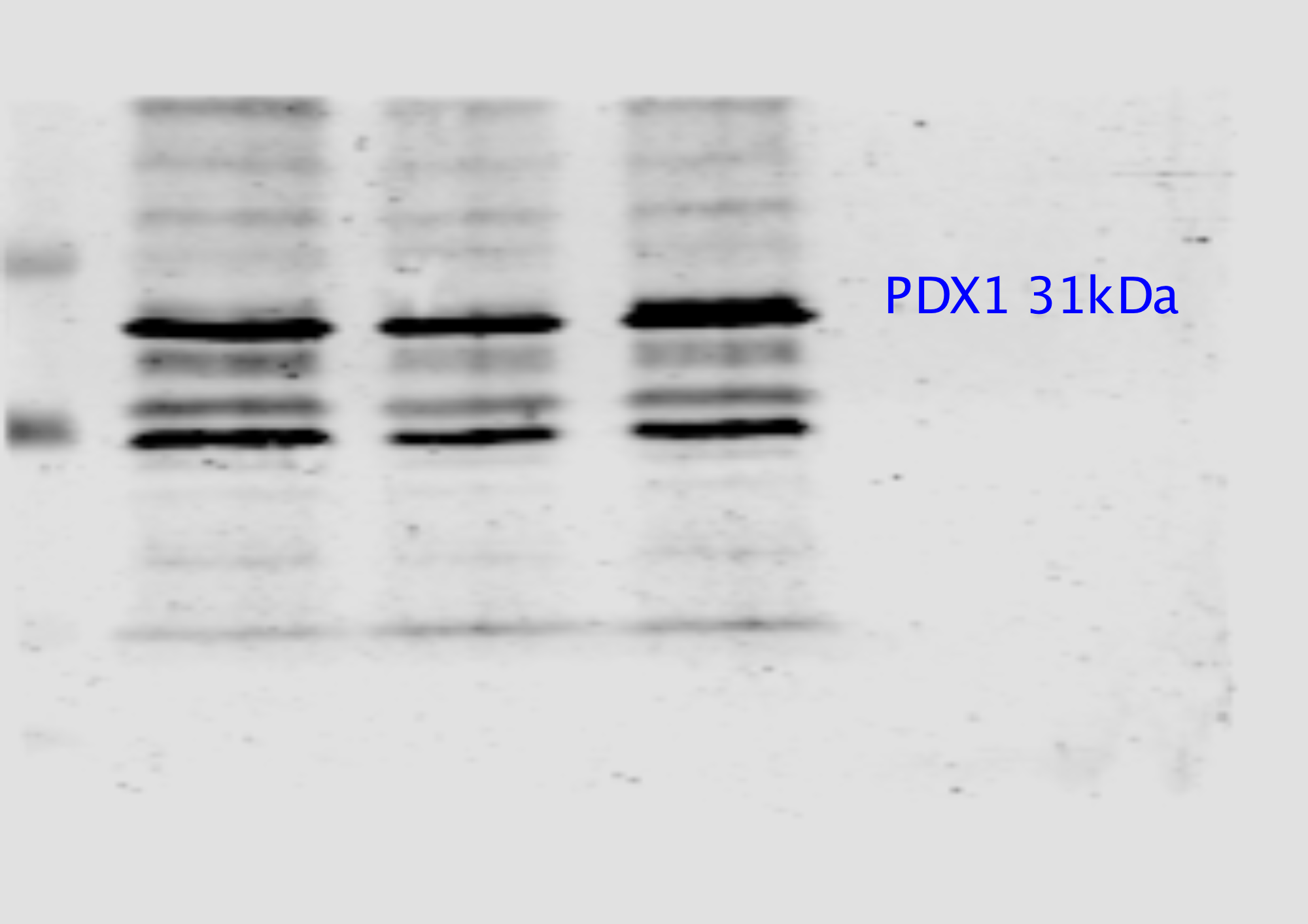

Supplement: Figure 2—source data 2. [file elife-83712-fig2-data2.zip › Figure 2- source data 2/unlabelled and uncropped/pdx1 rep2 in PDACB and BSNA9.4a and 9.5a second membrane vinculin control.tif]

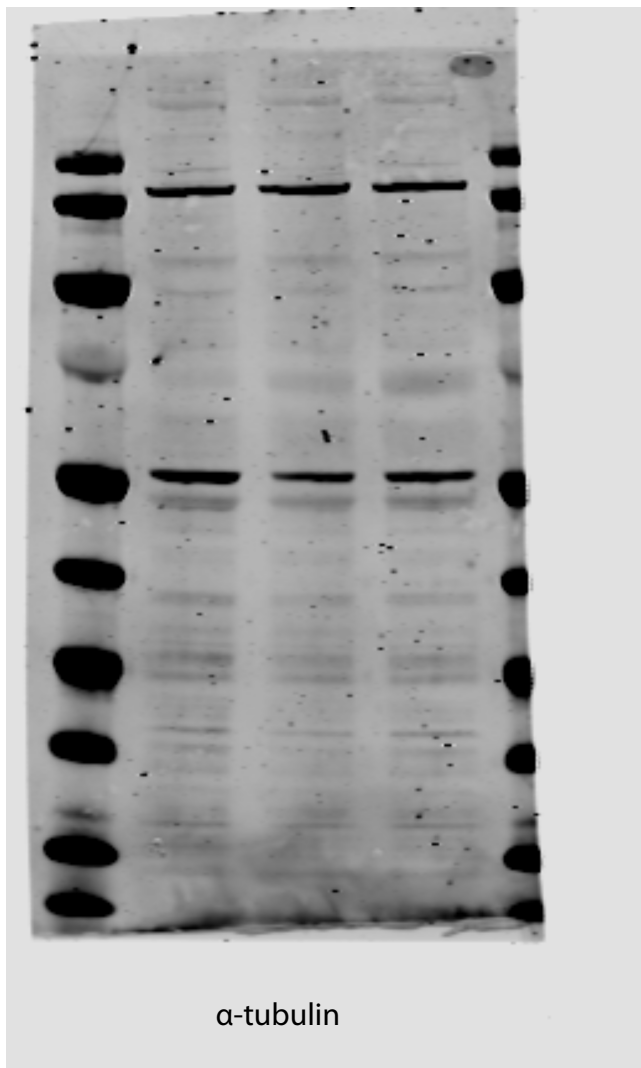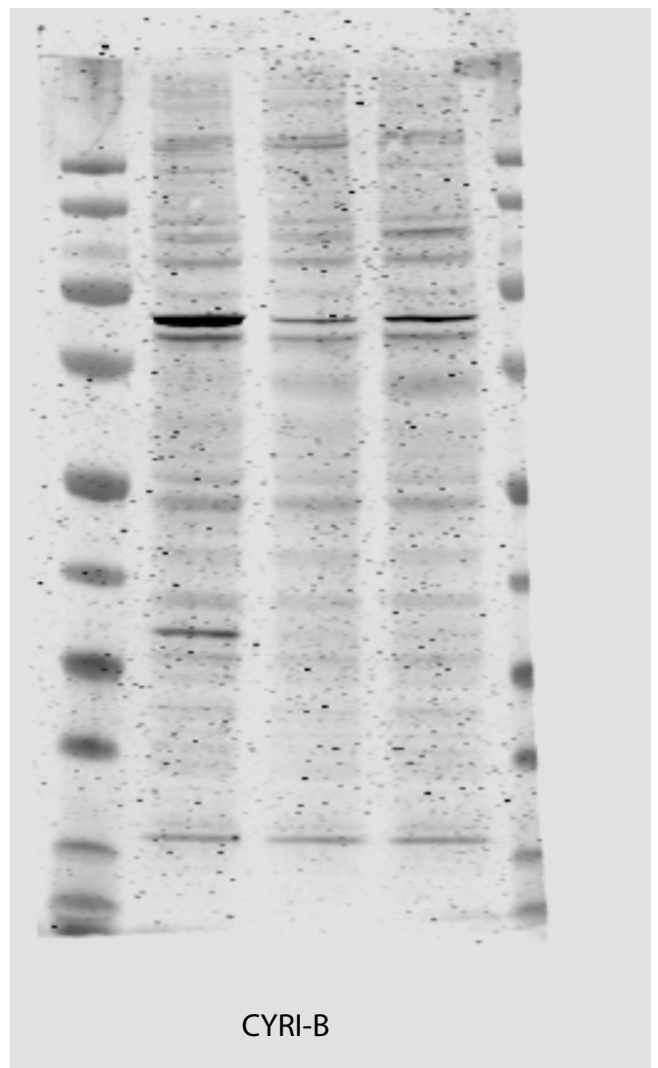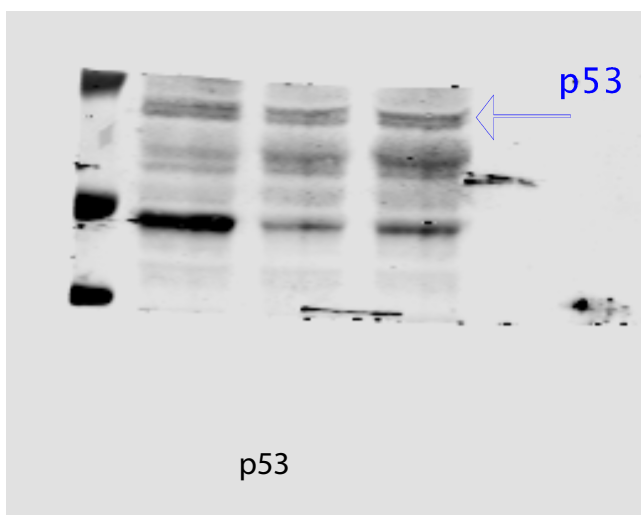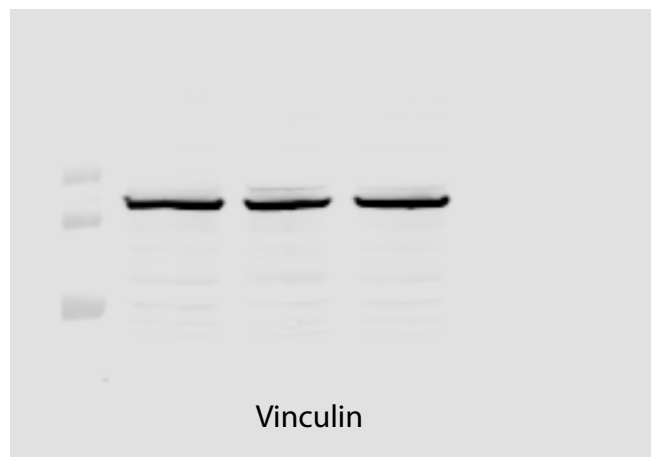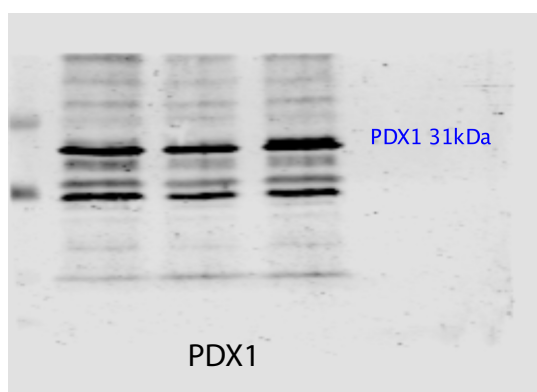

Supplement: Figure 2—source data 2. [file elife-83712-fig2-data2.zip › Figure 2- source data 2/unlabelled and uncropped/Unlabelled Figure 2D.pdf]

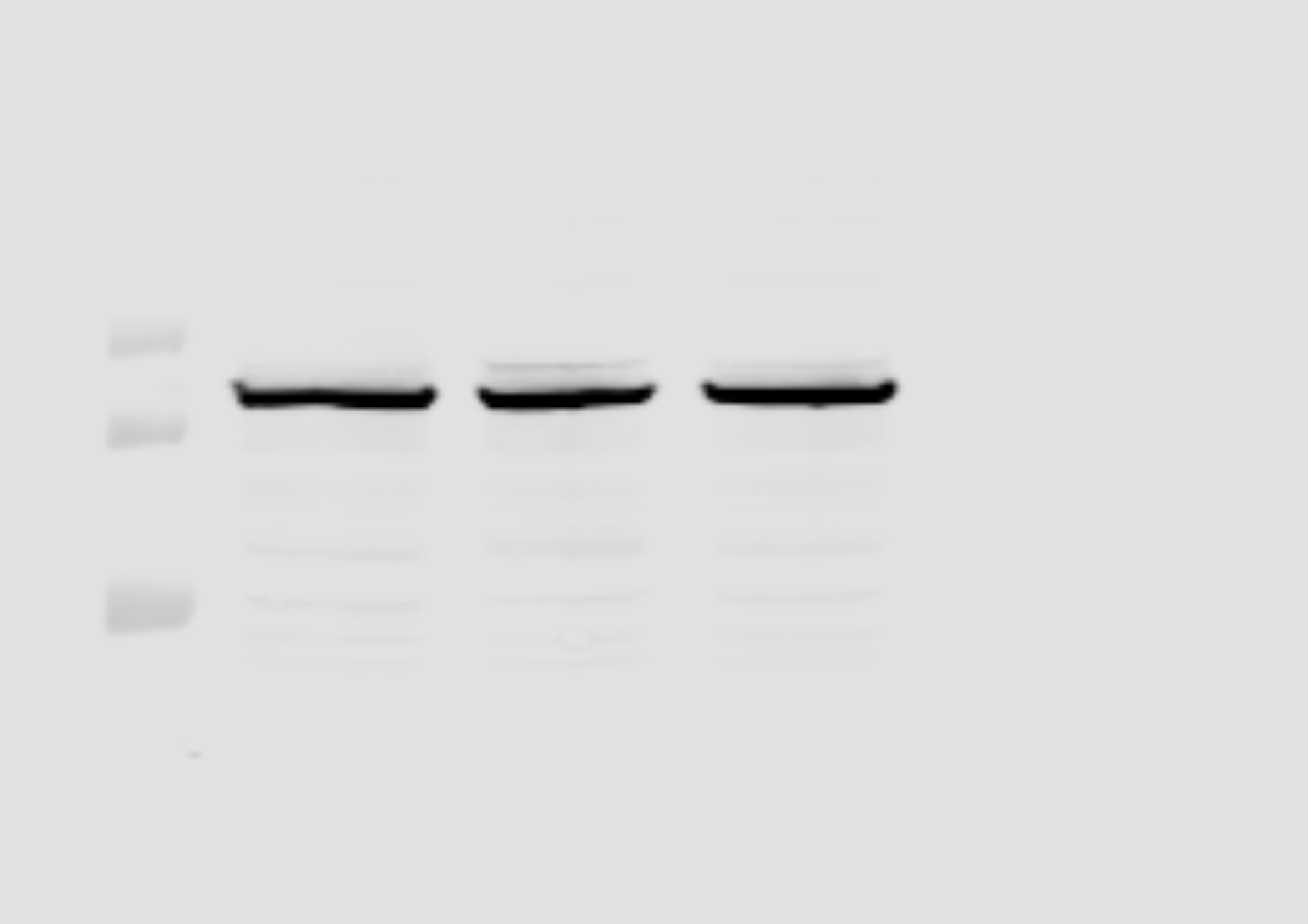

Supplement: Figure 2—source data 2. [file elife-83712-fig2-data2.zip › Figure 2- source data 2/unlabelled and uncropped/vinculin rep2 in PDACB and BSNA9.4a and 9.5a second membrane vinculin control.tif]

Figure 4- figure supplement 1A

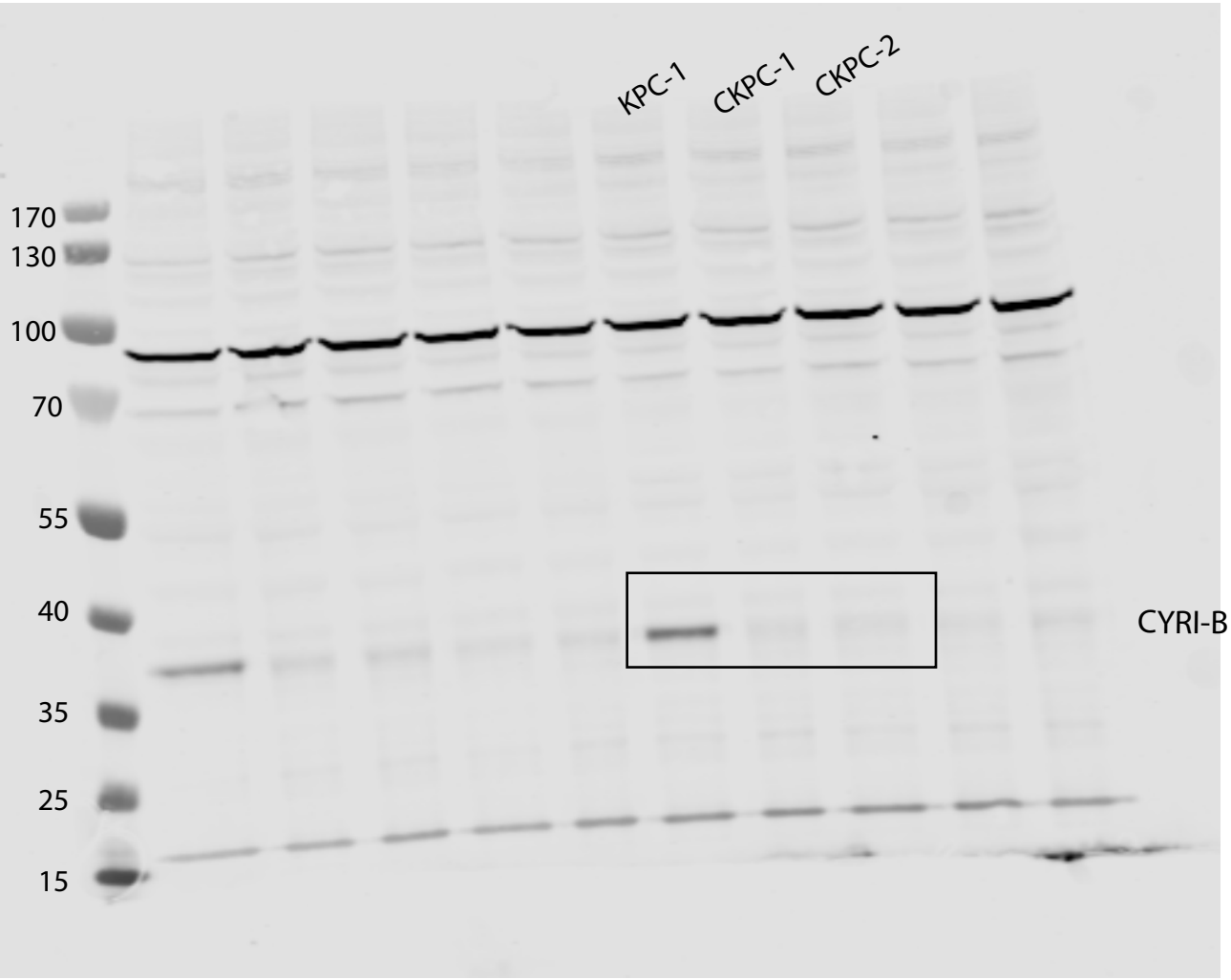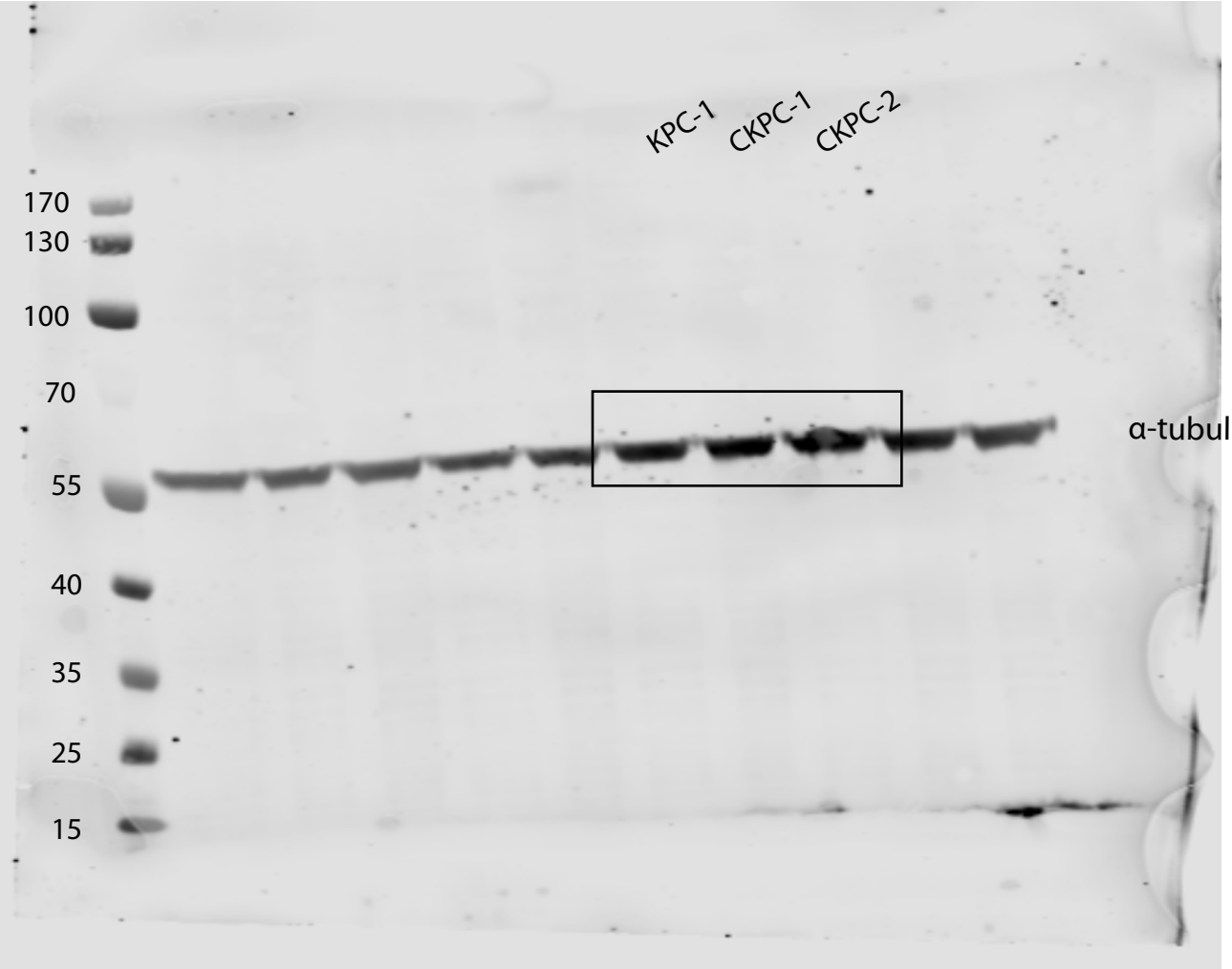

Supplement: Figure 4—figure supplement 1—source data 1. [file elife-83712-fig4-figsupp1-data1.zip › Figure 4- figure supplement 1- source data 1/Labelled/Figure 4- figure supplement 1.pdf]

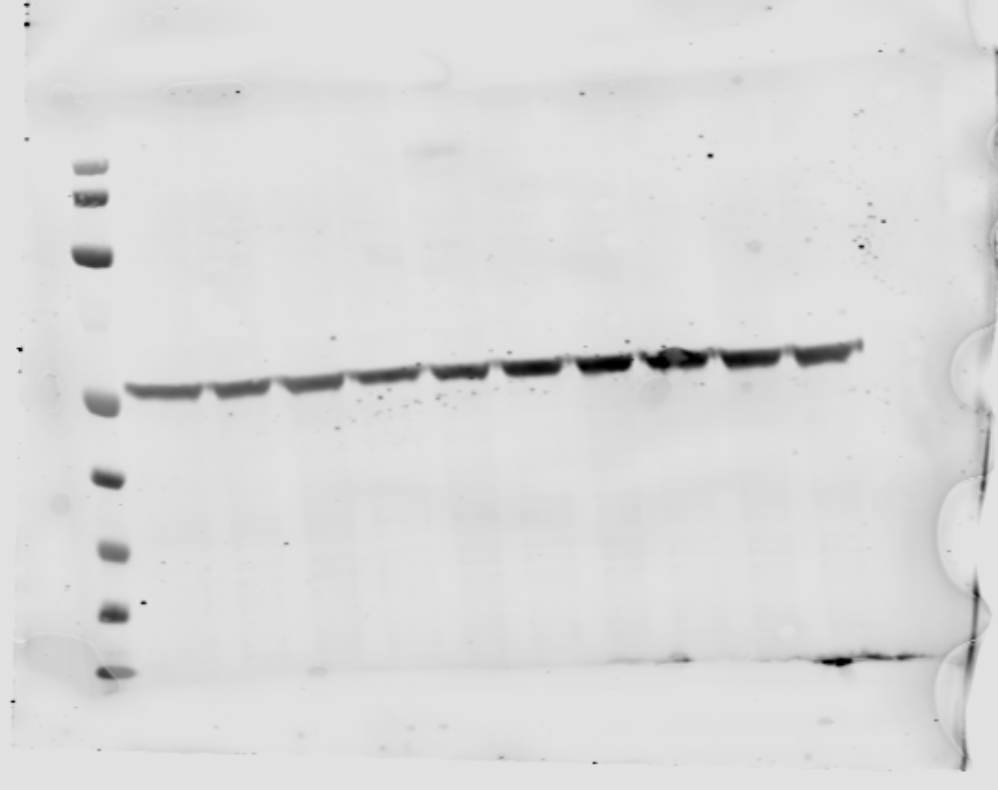

Supplement: Figure 4—figure supplement 1—source data 1. [file elife-83712-fig4-figsupp1-data1.zip › Figure 4- figure supplement 1- source data 1/Unlabelled and uncropped/a-tub stain in Fam49B KO 3rd week.png]

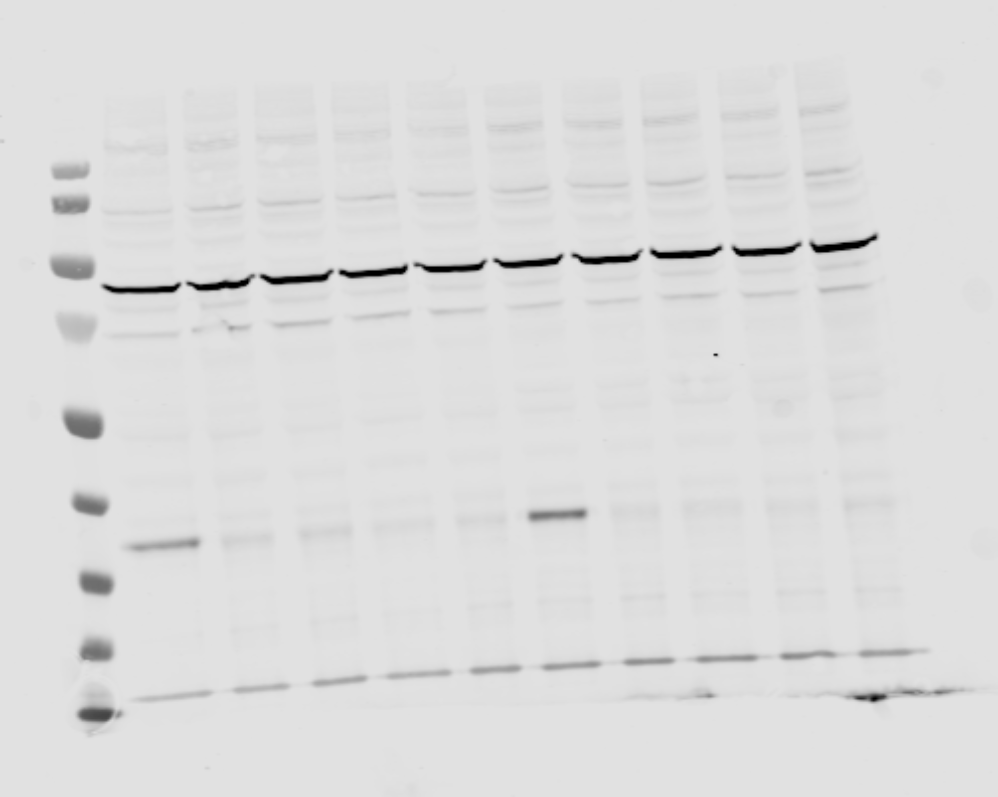

Supplement: Figure 4—figure supplement 1—source data 1. [file elife-83712-fig4-figsupp1-data1.zip › Figure 4- figure supplement 1- source data 1/Unlabelled and uncropped/Fam49B stain in Fam49B KO 3rd week.png]

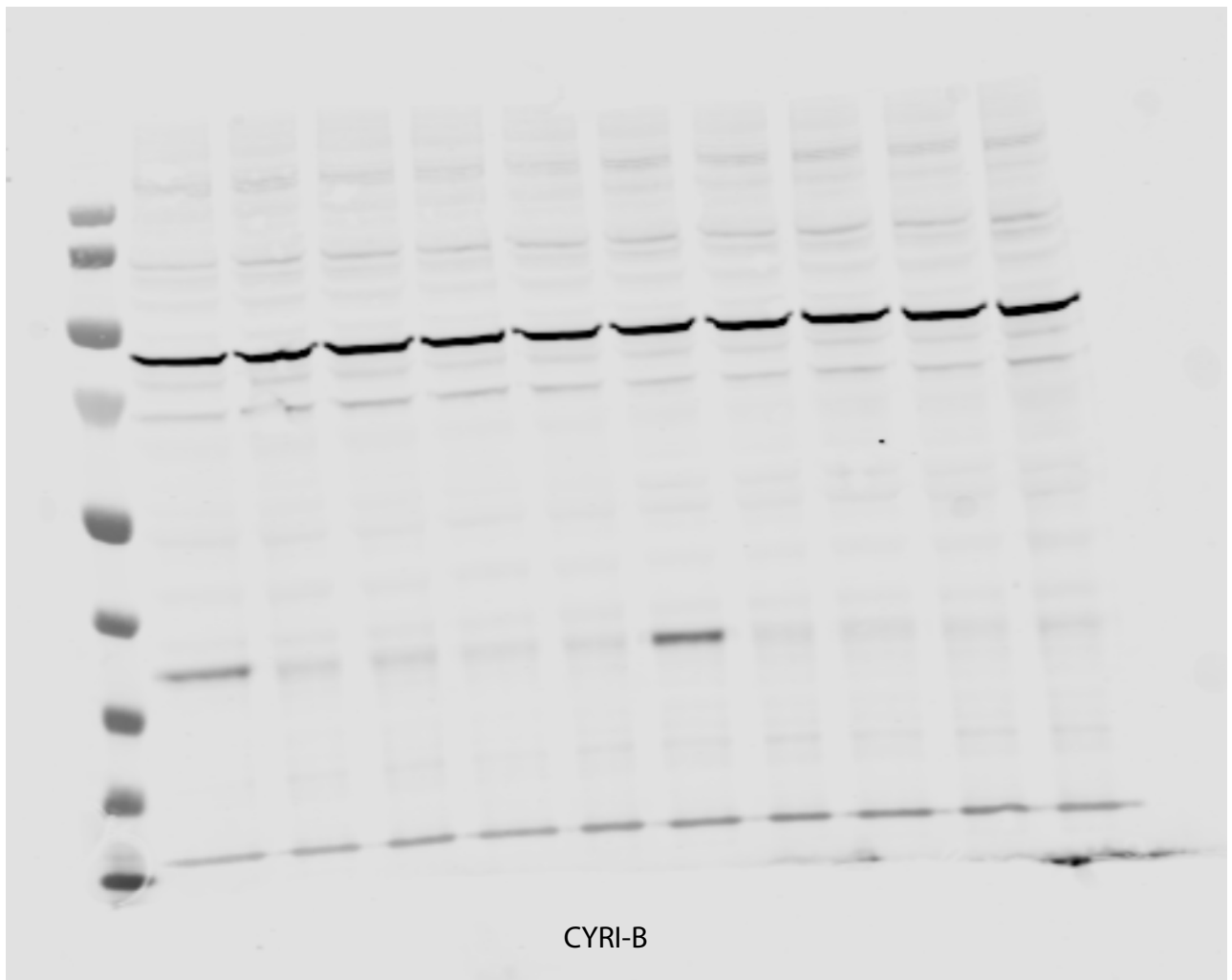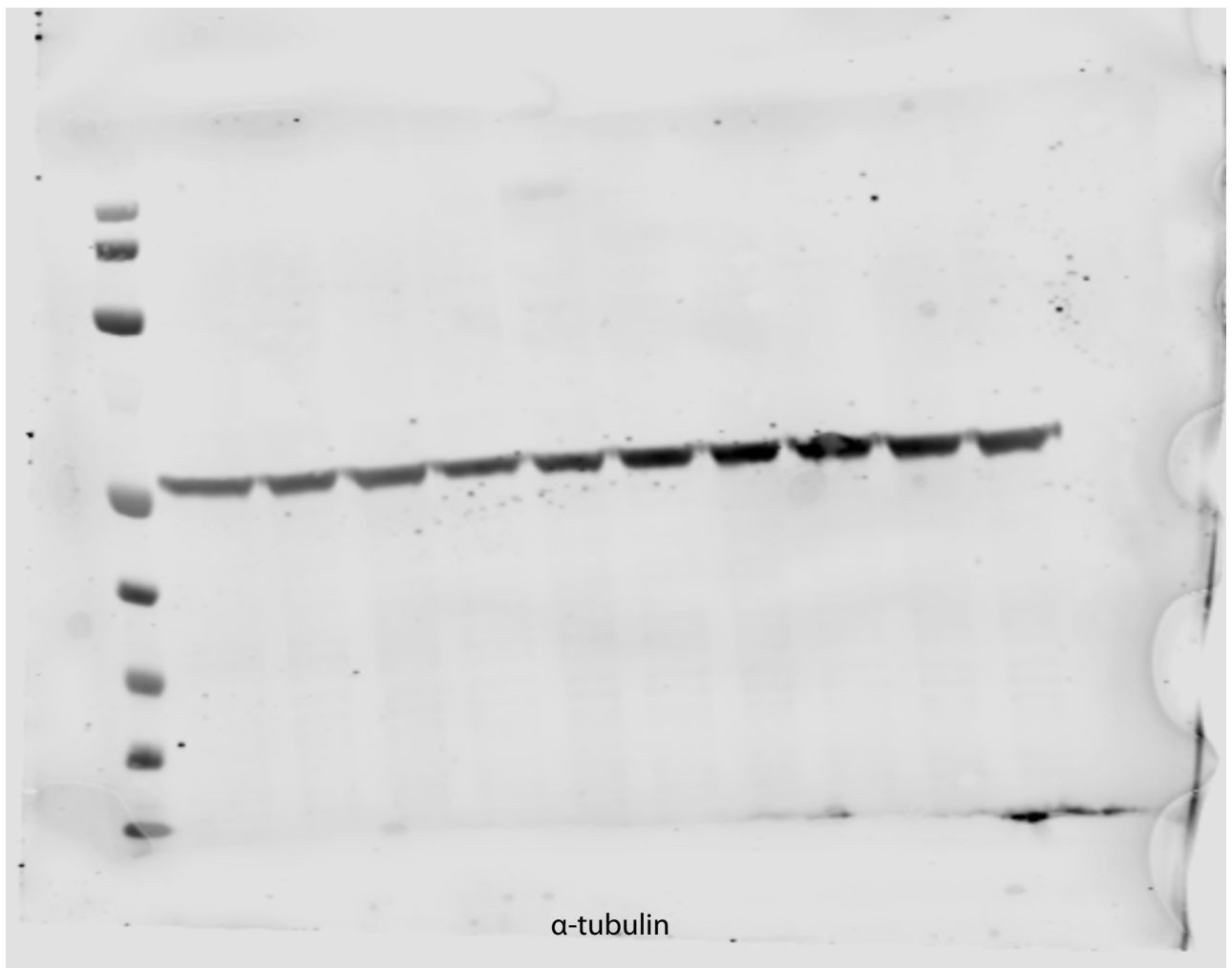

Supplement: Figure 4—figure supplement 1—source data 1. [file elife-83712-fig4-figsupp1-data1.zip › Figure 4- figure supplement 1- source data 1/Unlabelled and uncropped/Figure 4- figure supplement 1.pdf]

Figure 4- figure supplement 2A

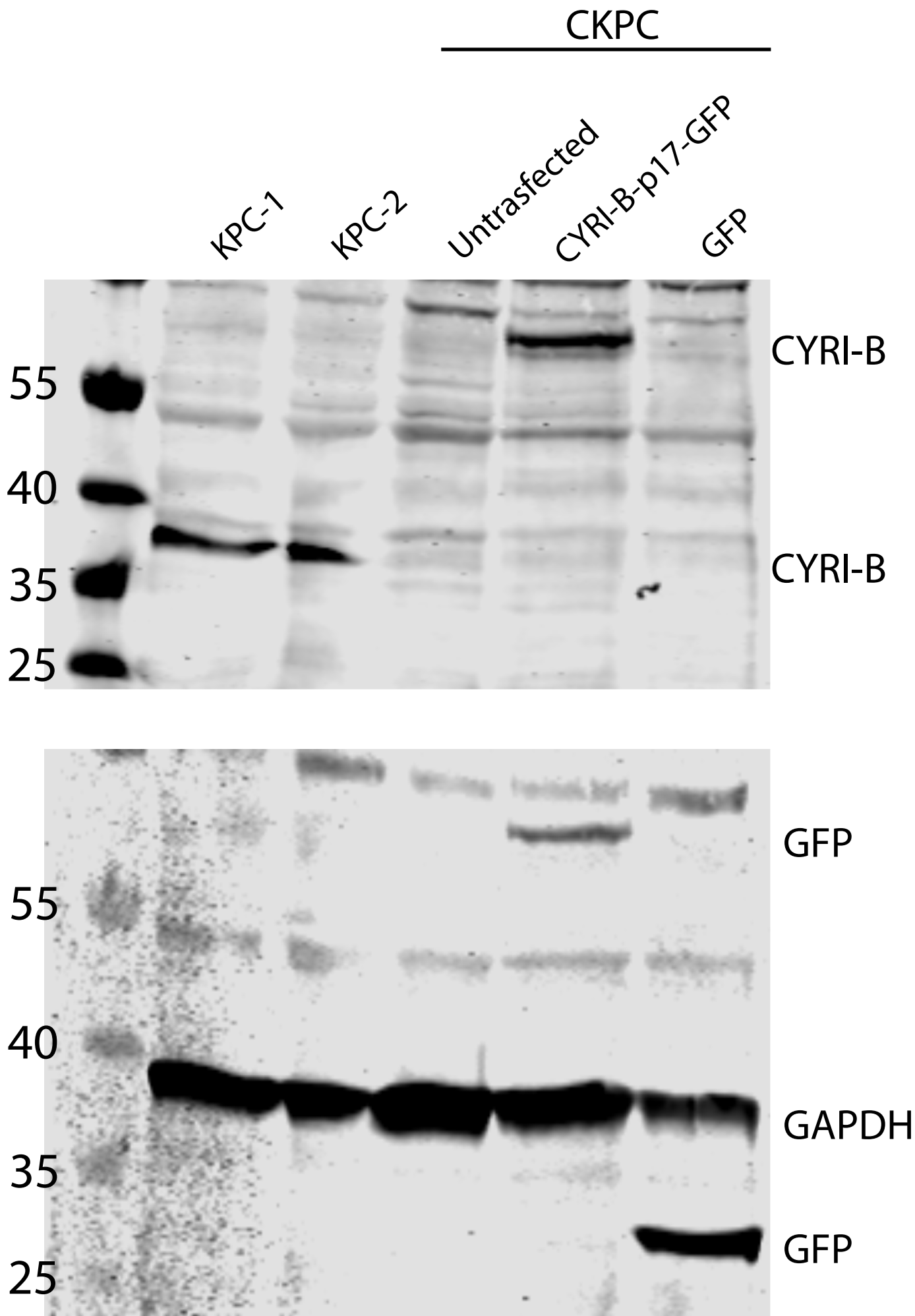

Supplement: Figure 4—figure supplement 2—source data 1. [file elife-83712-fig4-figsupp2-data1.zip › Figure 4- figure supplement 2- source data 1/labelled/Figure 4- figure supplement 2A.pdf]

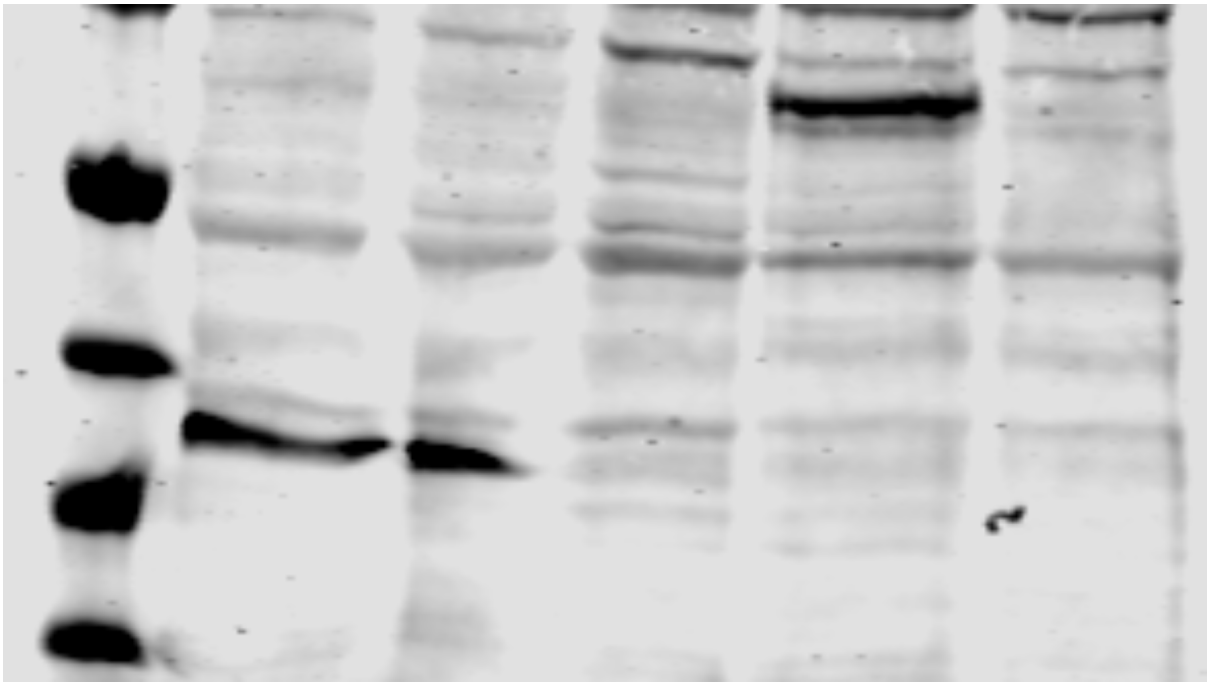

CYRI-B

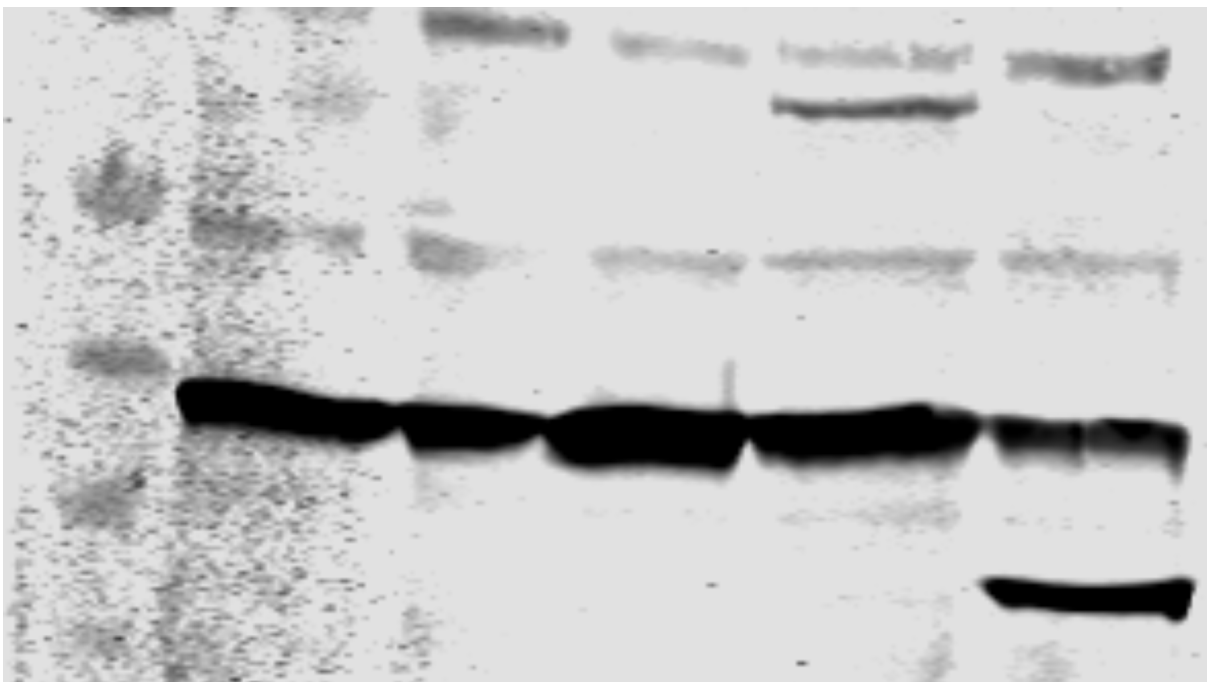

GFP and GAPDH

Supplement: Figure 4—figure supplement 2—source data 1. [file elife-83712-fig4-figsupp2-data1.zip › Figure 4- figure supplement 2- source data 1/Unlabelled and uncropped/Figure 4- figure supplement 2A.pdf]
