# Supplementary material for: CYRI-B-mediated macropinocytosis drives metastasis via lysophosphatidic acid receptor uptake
Source: eLife. 2024 May 7;13:e83712. doi: 10.7554/eLife.83712 (PMC11219039; doi:10.7554/eLife.83712)
Supplement: Figure 2—source data 3. — Excel data and Prism analysis to support Figure 2E. [file elife-83712-fig2-data3.zip › Figure 2 source data 3/Figure 2- source data 3 word 2E.docx]

Mouse counted in the survival curve are in the prism file next to the days and below:

| **WT:** | Time (Days) | WT | hom |
| --- | --- | --- | --- |
| BAJF91.1d | 133 | 0 |  |
| BSNA1.2a | 126 | 1 |  |
| BSNA7.1e | 95 | 0 |  |
| BSNA5.1e | 91 | 0 |  |
| BSNA7.2b | 72 | 0 |  |
| BSNA7.2d | 170 | 1 |  |
| BSNA7.2g | 130 | 1 |  |
| BSNA13.1e | 114 | 0 |  |
| BSNA14.1d | 159 | 0 |  |
| BSNA12.2b | 71 | 0 |  |
| BSNA10.3c | 71 | 0 |  |
| BSNA9.3c | 95 | 0 |  |
| BSNA15.3a | 125 | 0 |  |
| BAJF91.1a | 272 | 1 |  |
| BAJF97.1a | 146 | 0 |  |
| BAJF97.1b | 169 | 0 |  |
| BSNA35.1a | 125 | 1 |  |
| BSNA39.1c | 86 | 1 |  |
| BSNA5.2c | 296 | 1 |  |
| BSNA20.1d | 188 | 0 |  |
| BSNA28.1d | 102 | 0 |  |
| BAJF107.2b | 67 | 1 |  |
| BSNA43.2d | 92 | 1 |  |
| BSNA38.1d | 152 | 0 |  |
| BSNA32.5b | 102 | 1 |  |
| BSNA39.1f | 120 | 1 |  |
| BSNA14.1g | 167 | 1 |  |
| BSNA41.2d | 117 | 1 |  |
| BSNA31.2a | 186 | 1 |  |
| BAJF108.1f | 170 | 1 |  |
| BSNA35.1c | 205 | 1 |  |
| BSNA35.1g | 207 | 1 |  |
| BSNA34.2c | 180 | 1 |  |
| BSNA36.1d | 212 | 1 |  |
| BSNA41.1a | 207 | 1 |  |
| BSNA41.1d | 157 | 0 |  |
| BSNA31.3c | 98 | 0 |  |
| BSNA31.1b | 87 | 0 |  |
| BSNA41.3b | 64 | 0 |  |
| BSNA43.3a | 167 | 0 |  |
| BAJF108.1a | 139 | 0 |  |
| BAJF104.1d | 66 | 0 |  |
| BSNA43.3c | 204 | 1 |  |
|  |  |  |  |
|  |  |  |  |
|  |  |  |  |
|  |  |  |  |
|  |  |  |  |
|  |  |  |  |
|  |  |  |  |
| **HOM** |  |  |  |
| BSNA9.1d | 132 |  | 1 |
| BSNA8.1c | 220 |  | 0 |
| BSNA8.1f | 118 |  | 1 |
| BSNA12.1a | 103 |  | 1 |
| BSNA13.1b | 99 |  | 1 |
| BSNA8.2c | 117 |  | 1 |
| BSNA15.1a | 171 |  | 1 |
| BSNA11.1f | 179 |  | 1 |
| BSNA11.1g | 93 |  | 1 |
| BSNA9.4a | 70 |  | 1 |
| BSNA9.4d | 112 |  | 1 |
| BSNA10.4d | 97 |  | 1 |
| BSNA24.1c | 55 |  | 0 |
| BSNA24.1d | 173 |  | 1 |
| BSNA31.2c | 105 |  | 1 |
| BSNA19.1b | 195 |  | 1 |
| BSNA16.1a | 180 |  | 0 |
| BSNA11.3a | 104 |  | 1 |
| BSNA33.1c | 124 |  | 0 |
| BSNA37.2b | 156 |  | 1 |
| BSNA34.2i | 159 |  | 1 |
| BSNA38.1c | 92 |  | 0 |
| BSNA49.2c | 98 |  | 0 |
| BSNA58.1d | 100 |  | 1 |
| BSNA60.1d | 76 |  | 1 |
| BSNA56.1g | 136 |  | 1 |
| BSNA64.1h | 91 |  | 1 |
|  |  |  |  |
|  |  |  |  |
|  |  |  |  |
|  |  |  |  |
|  |  |  |  |
|  |  |  |  |
|  |  |  |  |
|  |  |  |  |
