# Supplementary material for: CYRI-B-mediated macropinocytosis drives metastasis via lysophosphatidic acid receptor uptake
Source: eLife. 2024 May 7;13:e83712. doi: 10.7554/eLife.83712 (PMC11219039; doi:10.7554/eLife.83712)

Image ID: 0000071_02
Acquire Time: 16-Sep-2020 13:58:12

Acquisition Information

#	Image ID	Acquire Time	Channels	Resolution	Intensities	Image Name	Comment	Image Modifications
1	0000071_02	16-Sep-2020 13:58:12	700 800	169um	Auto Auto	0000071_02		Crop Image ID: 0000071_01

Image Display Values

Channel	Color	Minimum	Maximum	K
700	Gray Scale (Black on White)	6.10	89.0	0.5

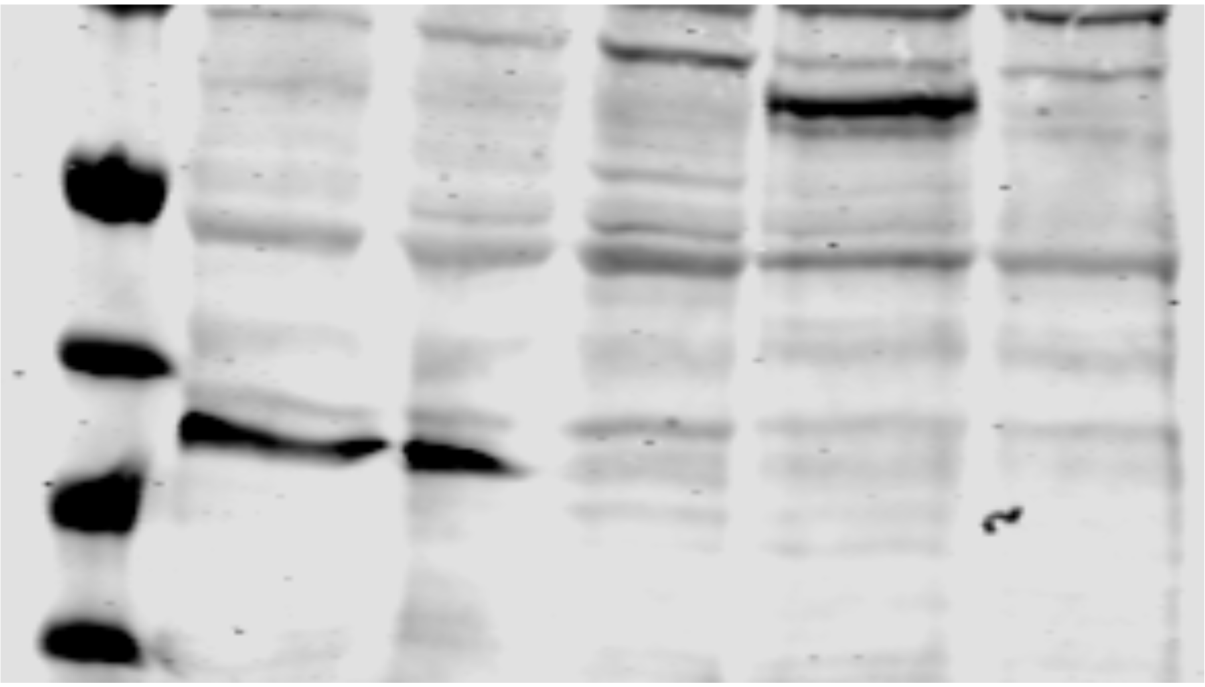

Supplement: Figure 4—figure supplement 2—source data 1. [file elife-83712-fig4-figsupp2-data1.zip › Figure 4- figure supplement 2- source data 1/Unlabelled and uncropped/Fam49B.pdf]
